# Reducing Social Disparity in Liver Transplantation Utilization through Governmental Financial Support

**DOI:** 10.5812/hepatmon.6463

**Published:** 2012-11-01

**Authors:** Kamran B. Lankarani, Mojtaba Mahmoodi, Siavash Gholami, Soheila Mehravar, Seyed Ali Malekhosseini, Sayed Taghi Heydari, Elham Zarei, Heshmatollah Salahi, Saman Nikeghbalian, Seyed Alireza Taghavi, Parisa Janghorban, Fariborz Ghaffarpasand

**Affiliations:** 1 Health Policy Research Center, Shiraz University of Medical Sciences, Shiraz, IR Iran; 2 Shiraz Organ Transplantation Center, Shiraz University of Medical Sciences, Shiraz, IR Iran; 3 Student Research Committee, Shiraz University of Medical Sciences, Shiraz, IR Iran; 4 School of Medicine, Jahrom University of Medical Sciences, Jahram, IR Iran; 5 Gastroenterology and Haepatology Research Center, Namazi Hospital, School of Medicine, Shiraz University of Medical Sciences, Shiraz, IR Iran

**Keywords:** Liver Transplantation, Financial Support, Iran

## Abstract

**Background:**

A high proportion of patients suffering from end stage liver disease are from low socioeconomic classes , which limits their access to liver transplantation as the most effctive treatment of this condition because of cost barrier.

**Objectives:**

one of the most challenging aspects of liver transplantation is its affordability and utilization by those who need it the most.

**Patients and Methods:**

Since November 2005, Iran Ministry of Health had covered 100% of the costs of in-patient liver transplantation care. To determine the effects of this policy, patterns of utilization of liver transplantation were compared before and after implementation of the policy. Group one included 112 and group two included 120 individuals who received transplantation before (from early January 2003 to November 2005) and after (from November 2005 to the end of December 2007) the legislation entered into the effect, respectively. Socioeconomic characteristics of these patients were evaluated by data collected about house and car ownership, education level, employment status, and place of residence.

**Results:**

Coverage of the costs allowed more illiterate and semiliterate people (P = 0.032) as well as more unemployed or unskilled workers to receive transplantation (P = 0.021). The number of transplantations also increased in children and geriatric age group. This legislation also led to greater countrywide regional coverage of indigent patients.

**Conclusions:**

This survey provides evidence that coverage of the costs by Ministry of Health was effective in reducing social discrimination in utilization of liver transplantation, and narrowed the gap between low and high socioeconomic classes in Iranian society.

## 1. Background

Disparities in the utilization of health facilities and the ways to tackle them are of great interest to policy makers in the health care sector. Despite the aim to channel resources toward those in need, it is not uncommon to see more usage among high socioeconomic sectors of societies throughout the world (1-3). Research in this field, although necessary and interesting, is challenging because such studies will usually necessitate major changes in national health policies and budgets. Liver diseases constitute one of the major causes of mortality and morbidity in Iran (4). With an approximate carriage rate of 3% for hepatitis B in middle age and elderly and a growing incidence of nonalcoholic steatohepatitis as well as increasing life expectancy, the number of people with chronic liver diseases is expected to rise in the future. According to a recent report by Iranian Ministry of Health, benign and malignant diseases of digestive tract and liver account for 8.3% of all non-accidental deaths, and next to cardiovascular diseases which are the leading cause of death (47%). Moreover, the incidence of several liver diseases is rising (5). Liver transplantation is the only effective treatment for most patients with liver failure (6). Aside from the costs of post-operative care and transplant-related medication, liver transplantation per se is an expensive procedure. The mean cost of a liver transplant is about US$163,438 (US$145,277–181,598) in the United States and about US$103,548 (US$85,514–121,582) (7) in other member states of organization for economic cooperation and development (OECD) countries. At Shiraz Organ Transplantation Center this cost is estimated at approximately US $38,000 (8). The relatively high number of patients in low-income sectors of society as well as high cost of the procedure itself my limit access of those with the greatest need to the procedure leading to potential social disparities in utilization of this treatment modality. The correlation between prevalence of chronic liver diseases and low socioeconomic status (SES) is well known (9). This factor is more prominent in developing countries where socioeconomic disparity restricts utilization of both traditional and modern health-care facilities in lower socioeconomic classes (10). In addition, compared to higher social classes, other risk factors of end-stage liver disease such as hepatitis B and C, and hepatocellular carcinoma are more prevalent in poor societies (11-13). Liver transplantation has been performed in the Islamic Republic of Iran since 1993. From 1993 to November 2005, the cost of liver transplantation was covered mostly by patients themselves. Starting from November 2005, Iranian Ministry of Health decided to cover 100% of the costs related to liver transplantation surgery. According to this new plan, , all surgery-related expenses were covered by the Ministry of Health for patients selected by transplant team based on clinical grounds . The organ allocation system was changed to the model for end-stage liver disease (MELD) scoring system 1.5 years before this legislation. Previously, a combination of the united network for organ sharing (UNOS) and child-turcotte-pugh (CTP) scoring system was employed to select patients for this procedure.

## 2. Objectives

In this study we compared socioeconomic characteristics of patients treated by liver transplantation; also the number of procedures before and after government financial support was introduced. Effect of new legislation on geographic distribution of the patients was also evaluated.

## 3. Patients and methods

### 3.1. Study Population and Protocol

The study was performed as a pre- and post-intervention face-to-face interview survey at Shiraz Organ Transplantation Center, Namazi Hospital, Shiraz University of Medical Sciences, Shiraz, Iran between September and December 2010. Approval was obtained from the institutional review board and ethics committee of Shiraz University of Medical Sciences before starting the study. All participants and/or their parents submitted their written informed consent. Individuals who consented were interviewed personally. Answering to all or any of the questions was optional for all participants. We used a convenience sampling method with participants drawn from all literate individuals who agreed to participate in this study. The questions were asked during face-to-face interviews in Persian language. The interviewer intervened only to clarify a question if required. No attempt was made to lead the respondents by suggesting answers in any manner. A total of 232 patients participated, and were divided in two groups. Group one included 112 and group two included 120 individuals who received transplantation before (from early January 2003 to November 2005) and after (from November 2005 to the end of December 2007) the legislation came into force, respectively. The only exclusion criterion was refusal of patient to participate in the study.

### 3.2. Data Collection Form

Socioeconomic characteristics of the participants were recorded on a data collection form with items for house and car ownership (as proxies for income), level of education, employment status, and place of residence. All questions were open-ended with options for multiple responses.

### 3.3. Statistical Analysis

All data are reported as proportions or the mean ± SD for 95% confidence intervals. Independent t-tests were applied to compare quantitative variables between the groups. The chi-squared test or Fisher’s exact test was used to compare qualitative variables such as employment, sex, etc. A P value less than 0.05 was considered statistically significant.

## 4. Results

**Figure 1:**
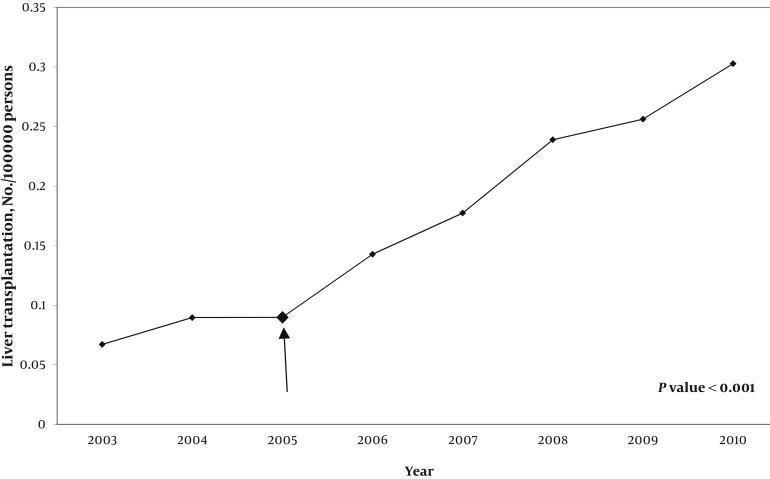
Number of Liver Transplantations in Iran Total number of liver transplantations performed at Shiraz Organ Transplantation Center per year from 2003 to 2010 (n = 1028). The arrow indicates starting time of governmental financial support covering 100% of the cost of transplantation.

**Figure 2:**
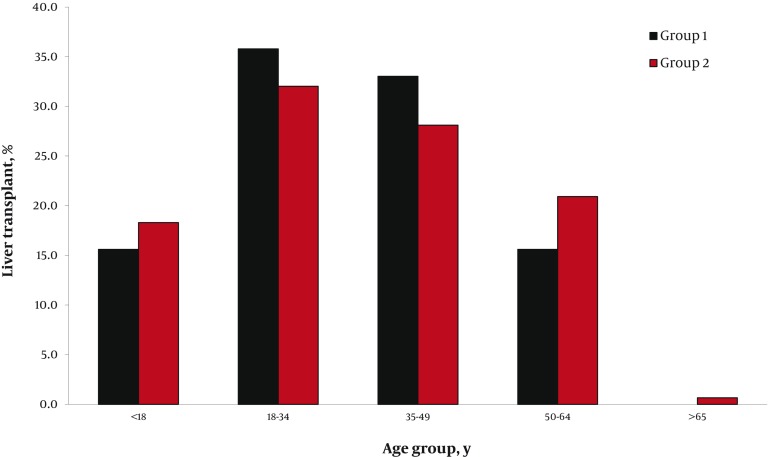
Age Distribution of the Recipients at the Time of Liver Transplantation Age distribution of the recipients before (Group 1) and after (Group 2) governmental financial support.

**Figure 3:**
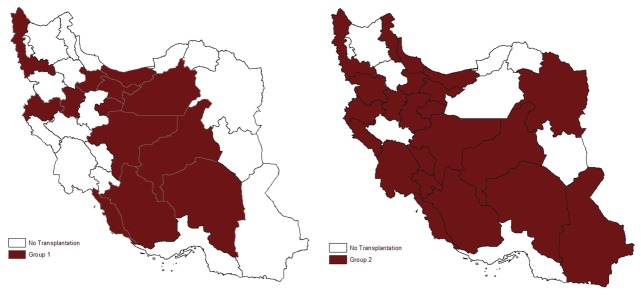
Geographical Distribution of Recipients by Province of Residence Geographical distribution of recipients before (Group 1) and after (Group 2) governmental financial support for liver transplantation

A total of 232 recipients were enrolled in two groups. In group one, 71 (64%) and in group two, 81 (67.5%) participants were female. After the law came into force, the number of transplantations performed per year increased dramatically (Figure 1). Although there was no substantial difference in the mean age of recipients before (33.5 ± 14.4 years) and after (30.3 ± 16.15 years) implementation of government law covering full costs of the procedure, the age distribution was changed (Figure 2). In group two, more patients in pediatric and geriatric age categories received liver transplants compared to group one, in which more middle-aged patients underwent transplantation. The two most commonly identified causes of end-stage liver disease in both groups were hepatitis B infection and autoimmune hepatitis (Table 1). Ascites in group one and jaundice in group two were the most common major complications. There were significant reductions in variceal bleeding, spontaneous bacterial peritonitis, and hepatic encephalopathy after commencement the law. However, appearance of jaundice as the most frequent complication of cirrhosis increased significantly (Table 2). After the beginning of ministerial coverage of transplantation costs, more illiterate and semiliterate people (P = 0.032) as well as more unemployed or unskilled workers received transplantation (P = 0.021). On the other hand, the proportion of participants who owned a house or a car increased slightly (Table 3). Assessment of geographical distribution in transplant recipients showed a greater achievement of regional coverage in group two (Figure 3). In place of residence terms, total number (30 in group one, 58 in group two) and variety of non-capital cities (18 in group one, 42 in group two) were increased in both groups.

**Table 1:** Etiology of the End-Stage Liver Diseases Among Liver Transplant Recipients in Iran, 2003 to 2007

	Group 1^a^, No. (%) (2003–2005)	Group 2^b^, No. (%) (2005–2007)	*P *value
**HBV infection **	26 (23.2)	21 (17.5)	0.114
**HCV infection **	6 (5.4)	4 (3.3 )	0.534
**HCC **	0 (0)	8 (6.7)	0.022 c
**Fulminant hepatitis **	1 (0.9)	1 (0.8)	0.999
**AIH **	19 (17)	19 (15.8)	0.999
**PBC **	1 (0.9)	1 (0.8)	0.999
**PSC **	16 (14.3)	8 (6.7)	0.016 c
**Crigler-Najjar syndrome **	0 (0)	7 (5.9)	0.044 c
**Budd-Chiari syndrome**	0 (0)	11 (9.2)	0.003 c
**Inherited metabolic liver diseases**			
Tyrosinemia	0 (0)	6 (5)	0.042 c
Wilson disease	11 (9.9)	9 (7.5)	0.360
Hemochromatosis	0 (0)	1 (0.8)	0.999
**Alcoholism**	2 (1.8)	1 (0.8)	0.574
**Biliary atresia**	4 (3.6)	4 (3.3)	0.999
**CHF **	1 (0.9)	0 (0)	0.419
**Caroli syndrome**	0 (0)	3 (2.5)	0.142
**Byler disease**	2 (1.8)	3 (2.5)	0.362
**Hypercholestrolemia**	0 (0)	3 (2.5)	0.267
**Cryptogenic cirrhosis**	22 (19.6)	5 (4.2)	0.291
**Overlap syndrome **			
AIH + PSC	0 (0)	2 (1.7)	0.267
**Combined diseases**			
HBV + PBC	0 (0)	1 (0.8)	0.999
HBV + HCV	1 (0.9)	2 (1.7)	0.267

Abbreviations: AIH, autoimmune hepatitis; CHF, congenital hepatic fibrosis; HBV, hepatitis B virus; HCC, hepatocellular carcinoma; HCV, hepatitis C virus; PBC, primary biliary cirrhosis; PSC, primary sclerosing cholangitis.

^a^Individuals who received transplantation after the new legislation.

^b^Individuals who received transplantation before the new legislation.

^c^Significant difference (P < 0.05).

**Table 2:** Major Complications of End-Stage Liver Diseases Among Liver Transplant Recipients in Iran, 2003 to 2007

	Group 1^a^, No. (%) (2003–2005)	Group 2 b, No. (%) (2005 – 2007)	*P* value
**Jaundice**	37 (33.3)	115 (74.7)	< 0.001 c
**Hepatic encephalopathy**	15 (13.5)	6 (3.9)	0.005 c
**Variceal bleeding**	17 (15.3)	4 (2.6)	< 0.001 c
**Ascites**	71 (64)	38 (36)	< 0.001 c
**SBP **	6 (5.4)	2 (1.3)	0.072
**Pulmonary hepatic failure**	0 (0)	2 (1.3)	0.511

Abbreviation: SBP, spontaneous bacterial peritonitis.

^a^Individuals who received transplantation before the new legislation.

^b^Individuals who received transplantation after the new legislation.

^c^Significant difference (P < 0.05).

**Table 3:** Demographic Characteristics of Liver Transplant Recipients in Iran, 2003 to 2007

	Group 1^a^, No. (%) (2003–2005)	Group 2 b, No. (%) (2005–2007)	*P* value
**Dwelling**			
Rented	20 (18.2)	18 (15)	0.874
Owned	90 (81.8)	102 (85)	0.874
**Car ownership**			
Yes	36 (31.9)	39 (32.8)	0.357
No	77 (68.1)	80 (67.7)	0.357
**Occupation**			
Unemployed	5 (4.4)	24 (20)	0.021 c
Farmer, gardener or unskilled worker	8 (7.1)	29 (24.1)	0.021 c
Teacher, professor or clerk	28 (25)	23 (19.1)	0.021 c
Shopkeeper or merchant	22 (19.6)	8 (6.7)	0.021 c
Student	24 (21.4)	12 (10)	0.021 c
Housekeeper	25 (22.3)	24 (20)	0.021 c
**Education**			
Illiterate	5 (4.4)	24 (20)	0.032 c
Semiliterate	4 (3.5)	54 (45)	0.032 c
High school graduate	66 (58.9)	32 (26.7)	0.032 c
University degree	37 (33)	10 (8.3)	0.032 c
**Area of residence (Rural or urban)**			
Seven largest cities	70 (62.5)	31 (25.8)	0.058
Other provincial capitals	12 (10.7)	31 (25.8)	0.058
Small cities or rural areas	30 (26.8)	58 (48.4)	0.058

^a^Individuals who received transplantation before the new legislation.

^b^Individuals who received transplantation after the new legislation.

^c^Significant difference (P < 0.05).

## 5. Discussion

Among non-communicable diseases, end-stage liver disease is one of the most important causes of death. In the last decade Iran has achieved great advances in extending access to liver transplantation facilities (14). Liver transplantation is a costly procedure which imposes a heavy economic burden on either recipients or governments as a health provider (15). Growing social inequities in health status in developing and developed countries, together with increasing inequities in income and wealth, draw attention to socioeconomic class as a key determinant of accessibility and utilization of health care facilities (10, 16). The terms “socioeconomic status”, “socioeconomic position” and “social class” are extensively used in health research, indicating widespread although often implicit recognition of the importance of socioeconomic factors for different health outcomes and estimations (17, 18). However, social class is a complex entity to measure. A number of factors are used as surrogate markers for social class, such as neighborhood income, insurance and education (19). In the United States of America, neighborhood income is used as a surrogate marker of actual income since disposable income for people with certain income levels can be limited because of property values (16). Reliable information on actual or estimated income is difficult to be obtained in Iran. Most people prefer to avoid talking about their salary or other income resources. Perhaps education, occupation, and house ownership are the best surrogate indicators of socioeconomic status in Iran. Our findings clearly indicate that the introduction of governmental financial support led to selection of more illiterate and semiliterate people as well as unemployed or unskilled workers for coverage of liver transplantation. These groups are among those who are most likely to be discriminated during the selection process. The same may occur in elderly and pediatric age groups, who were received more transplants after financial support program, too. Both these age groups are widely known to be vulnerable to discrimination.

Geographic location (metropolitan versus small cities, urban versus rural) is also one of the factors that may lead to discrimination in the allocation of organs for transplantation. This factor not only affects patients’ access to healthcare facilities, but may also be an indirect indicator of social class. We showed that after commencement of financial aid program, more patients from small cities and rural areas received liver transplantation (Table 3). Furthermore, cases from more diverse areas of the country were selected for liver transplantation (Figure 3). Another result that is worth attention is the fact that after financial intervention, clinical presentation of transplantation candidates at the time of selection shifted from more serious and life-threatening conditions (variceal bleedings, hepatic encephalopathy) to less serious ones (jaundice). This may be the result of increased number of transplantations which made the selection of patients with less severe liver disease possible in conjunction with those who suffer from more advanced disease. Some surveys have considered social disparity in the context of liver transplantations with regard to different aspects such as racial and insurance disparities (20), racial disparities in transplantations for hepatitis B (21), disparities among blacks and whites (22), and disparities related to hepatocellular carcinoma (23). To date, however, it appears that no comprehensive studies focused on the utilization of liver transplantation facilities by high and low socioeconomic classes. The approach used for socioeconomic evaluation in our analysis might be a potential limitation. However, it should be emphasized that accurate determination of g socioeconomic status in developing countries such as Iran is not an easy task. For example, Yoo et al. (24) applied Hollingshead Index of Social Status to identify and quantify socioeconomic status. But this classification, which is based on education and occupation, is not completely applicable in Iran, and its validity, reliability, and efficacy could not be verified with translated version of this index. Another way to evaluate SES used by Yoo et al. (25) was based on neighborhood income, education, and insurance. This way is not practical in Iran because of absence of zip codes, considered as a reliable source of information for median income. We therefore concluded that it was necessary to apply available measures and variables in Iran which are reliable in terms of information they provide.

Lack of long-term follow-up of patients was another limitation of our survey. One of the most important aspects of a postoperative course in recipients of liver transplant is their survival. Nonetheless, the impact of SES on survival remains controversial. As access to liver transplantation increases, additional researches in regard to long-term follow-up will be needed to assess whether financial support such as that provided by current Iranian system has any impact on long term survival of patients with low SES and end stage liver disease. In Iran before governmental sponsorship, patients had to pay costs of surgery from their own pocket, except very few cases whom were covered by semi-private insurances. After availability of government-mandated coverage, all patients had the same access to liver transplantation facilities. As a result of rising number of liver transplantations, Ministry of Health was obliged to support higher costs of post-transplantation medication. Government subsidies and insured medication costs led to dramatic decreases in the costs of drugs. Through such financial support complex, patients now pay only 3% to 5% of total post-transplantation medication value. Our experience showed that provided financial support has been effective in reducing social discrimination in utilization of liver transplantation, and has narrowed the gap between low and high socioeconomic classes in Iran. We also showed that the total number of transplantations and access across the entire population of the country increased after public financial support became mandatory.
